# Review of epidemiologic data on the debate over smokeless tobacco's role in harm reduction

**DOI:** 10.1186/1741-7015-7-61

**Published:** 2009-10-19

**Authors:** David S Timberlake, Jason A Zell

**Affiliations:** 1 Program in Public Health, University of California, Irvine, Irvine, CA 92697, USA; 2 Department of Epidemiology, University of California, Irvine, Irvine, CA 92697, USA; 3 Division of Hematology/Oncology, Department of Medicine and Chao Family Comprehensive Cancer Center, University of California, Irvine, Orange, CA 92868, USA

## Abstract

Some tobacco researchers have argued that the European Union should remove its ban on a form of low-nitrosamine smokeless tobacco referred to as Swedish 'snus'. This argument has developed in to an international debate over the use of smokeless tobacco as a measure of harm reduction for smokers. Leading authorities in the USA have firmly stated that there is no safe tobacco - a message which does not allow for any discussion of comparative tobacco risks. This commentary is intended to review the origin of the controversy over Swedish 'snus', to examine briefly the meta-analysis on cancer risks by Peter Lee and Jan Hamling (published in July in *BMC Medicine*) and to discuss the anticipated direction of the debate on tobacco-harm reduction in the USA. We anticipate that much of the debate will shift from the discussion of epidemiologic data to the discussion of the marketing, health communication and economics of smokeless tobacco. While the Food and Drug Administration's newly approved authority over tobacco will undoubtedly affect the smokeless products, it may not be the sole determinant of harm reduction's fate in the USA.

See associated research article by Lee and Hamling:

## Origin of the controversy

The controversy over smokeless tobacco, considered by some as a potential substitute for cigarettes, originates from epidemiologic studies in Sweden - a country which has one of the lowest rates of daily smoking in Europe in recent years [1]. This achievement in tobacco control was particularly remarkable because of the significant gender difference in the smoking rates, leading one expert to question whether an experiment in harm reduction was underway [2]. The decline in daily smoking over the past two decades was more pronounced in Swedish males than in females [3,4], resulting in a lower prevalence by 2001 (15% versus 19%, respectively) [5]. This steep decline coincided with Swedish males' increased use of 'snus', a form of moist smokeless tobacco which harbours fewer cancer-causing nitrosamines than its American counterparts [6]. Furthermore, the apparent substitution of tobacco products by the Swedish males coincided with a decrease in the incidence of lung cancer, a trend which has not been observed in Swedish females [7]. It is unlikely that existing tobacco-control policies could account for the difference in the prevalence of both smoking and smoking-related morbidity. Yet it is important to note that virtually all of the Swedish data supporting the cause of harm reduction is based on observational study designs, notably the cross-sectional design [3,5,8,9]. Thus, it is premature to state that the increased use of 'snus' is causally associated with tobacco substitution and the decline in morbidity. Emerging clinical trials are investigating smokeless tobacco as a tool for smoking cessation [10]. In one randomized clinical trial of smokeless-tobacco use in combination with group support (versus group support alone), smoking rates decreased at the end of the 7-week period for the intervention group [11]. This effect, however, was not maintained after 6 months.

The current debate over harm reduction has focused primarily on two epidemiologic issues. The first relates to the cancer risk from the use of smokeless products, the basis for our commentary on the article by Peter Lee and Jan Hamling [12]. The second issue addresses whether use of smokeless tobacco is associated with the initiation or cessation of smoking. These issues have increasingly been investigated in the USA where smokeless tobacco is frequently marketed. Data, based on a single cross-sectional study [13], suggests that smokers in the USA are exchanging their cigarettes for smokeless tobacco. However, a longitudinal study of USA residents [14] reported that only 0.3% of male smokers changed to smokeless tobacco compared to 3.9% of male smokeless-tobacco users who subsequently changed to smoking tobacco.

The issue concerning the transition from smokeless tobacco to smoking raises the specter of the gateway effect. This issue has attracted much attention because of the belief that non-smoking adolescents could be unduly influenced to use a form of smokeless tobacco ('snus') intended for use by established smokers. A gateway effect, which could consequently lead to an adolescent's uptake of smoking, has been reported in some USA reports [15-17] but refuted in others [18,19]. One opposing argument states that the underlying risk factors for smoking, which tend to be greater in users of smokeless tobacco, account for the observed association between the tobacco products. This hypothesis was the basis for our use of the propensity score [20] in matching the users and non-users of smokeless tobacco [19]. Smokeless tobacco was a significant risk factor for smoking in this longitudinal study, but only prior to the matching of individuals on the propensity score. Even if a gateway effect to smoking exists, which is doubtful, only a minority of smokeless-tobacco users would be affected. Analyses of two national surveys indicate that less than 40% of smokeless-tobacco users in the USA had initiated use prior to the onset of smoking [21,22]. The remainder had either never initiated smoking or had smoked prior to their initial use of smokeless tobacco.

## Commentary on an article by Peter Lee and Jan Hamling

A systematic review by Lee and Hamling provides a comprehensive assessment of the state of evidence on the relationship between smokeless tobacco and cancer in Europe and North America [12]. Carcinogens in smokeless tobacco include high levels of nitrosamines, polycyclic aromatic hydrocarbons and other agents [23], underscoring the biologic relevance of this type of analysis. The primary findings of Lee and Hamling are that oropharyngeal cancers, in addition to prostate cancer (an association that may be spurious as it lacks biologic plausibility and is unsupported by prior research), were the only malignancies significantly associated with smokeless-tobacco use [12].

Researchers in cancer prevention refer to oropharyngeal cancer (along with cancers of the lung, esophagus and stomach) as an aerodigestive malignancy, a heterogeneous group of epithelial tumours affected by 'field carcinogenesis' from a common exposure - inhaled tobacco smoke. Beyond alcohol and tobacco smoking, recent evidence has implicated a new aetiologic factor for a subset of oropharyngeal and laryngeal cancer patients: human papilloma virus (HPV) [24]. In contrast to other oropharyngeal cancers, HPV-associated oropharyngeal cancers occur in younger patients and are characterized by an equal gender distribution, a lower tumour grade of differentiation, less association with alcohol and tobacco use and improved survival outcomes. The relevant oropharyngeal cancer-associated HPV serotypes (HPV 16, 18, others) and mode of transmission (sexual) are common to cervical cancer. Clearly, HPV-status was not reported in the individual studies reviewed in this meta-analysis, but smokeless-tobacco use has been associated with young age [25] and risky behaviour (including youth rebelliousness and pressure to be sexually active) [26]. Although HPV status is a potential confounder for the reported association of smokeless tobacco with oropharyngeal cancer, it is unlikely to explain the excess risk observed in the Lee and Hamling article - an effect that is consistent with other reports [25].

This lack of association of smokeless-tobacco use with cancers of the lung, esophagus, stomach, pancreas, bladder, kidney and haematologic malignancies starkly contrasts with research published over the past six decades on smoking-related cancer risks [27-29]. Despite the observational nature of these results, the overwhelmingly null associations with cancer in this high quality analysis are provocative, if not compelling. It is important to note that these results are not entirely congruent with prior reports on smokeless-tobacco use which demonstrate an increased risk of oral, esophageal and pancreas cancers [25]. Potential reasons for the apparent discrepancies are discussed by Lee and Hamling in a separate paper [30].

## Future directions

Attention in the coming years is likely to shift from an epidemiologic debate to a debate on the marketing, health communication and economics of the smokeless products. In Europe, researchers and policymakers will continue to debate the European Union's ban on 'snus' [31]. In the USA, attention will most likely be focused on the tobacco industry's marketing of the 'snus'-like products. This, of course, will depend greatly on the Food and Drug Administration's (FDA) newly approved regulation of the tobacco industry [32]. A historical examination of tobacco documents reveals that the Philip Morris company had anticipated smoking restrictions and the benefits of smokeless products as early as 1984 [33]. As a consequence, cigarette manufacturers have invested resources into the smokeless-tobacco market.

A question of primary interest is whether the tobacco companies will market 'snus' (for example, Camel Snus) for harm reduction, given FDA approval, or market the tobacco as a situational substitute for the smokers who frequently encounter smoking restrictions. Carpenter *et al.*'s examination suggests that the industry is more interested in the dual use of tobacco products, rather than tobacco substitution [33]. A scenario of graver concern is that adolescents will use 'snus' because the tobacco is misperceived as being safe. The low level of nicotine delivery in Marlboro Snus [34], a level insufficient for supplanting cigarettes, elicits memories of the graduation strategy [35] and the related manipulation of the 'free' nicotine content of moist snuff [36]. It is yet to be determined when and if the FDA will regulate the nicotine yields of the smokeless products. The FDA will, however, mandate that tobacco companies provide sufficient evidence of harm reduction before such a claim can be stated publically [37]. This position reflects the sentiment of other state and federal agencies (for example the US Center for Disease Control and Prevention) which are reluctant to disseminate information on comparative tobacco risks. This reluctance may be in response to the wide variation in toxicant levels of smokeless products [38]. Others have argued, however, that such a position is a violation of smokers' rights to be given accurate information [39], and may be a factor which could account for the preponderance of misperceived tobacco risks [40-43]. Risk perceptions aside, most smokers are not receptive to using smokeless tobacco as a substitute, a finding reported in the 2005 California Tobacco Survey [44]. Thus, factors other than the FDA's oversight, such as a cultural influence on 'snus' use [45], may ultimately determine the fate of tobacco-harm reduction in the USA.

## Abbreviations

CDC: Centers for Disease Control and Prevention; FDA: US Food and Drug Administration; HPV: human papilloma virus.

## Competing interests

The authors declare that they have no competing interests.

## Authors' contributions

DT reviewed articles on harm reduction and authored the sections of the manuscript entitled 'Origin of the controversy' and 'Future directions'. JZ reviewed articles on cancer risks and authored the section entitled 'Commentary on article by Peter Lee and Jan Hamling'.

## Pre-publication history

The pre-publication history for this paper can be accessed here:


